# Knowledge of and Attitude Toward Preventive Care Among Iraqi Dentists and Senior Dental Students: A Cross-Sectional Study

**DOI:** 10.7759/cureus.65120

**Published:** 2024-07-22

**Authors:** Bassam Alsheekhly, Mahshid Namdari, Maha Jamal Abbas, Hadi Ghasemi

**Affiliations:** 1 Department of Community Oral Health, School of Dentistry, Shahid Beheshti University of Medical Sciences, Tehran, IRN; 2 Department of Epidemiology, School of Public Health and Safety, Shahid Beheshti University of Medical Sciences, Tehran, IRN; 3 Department of Orthodontics, Pedodontics and Preventive Dentistry, College of Dentistry, Mustansiriyah University, Baghdad, IRQ

**Keywords:** dentists, preventive dentistry, dental students, attitude, knowledge

## Abstract

Objectives: Acquiring knowledge and maintaining a positive mindset are essential for dental care providers to offer optimal dental services. Dental professionals are expected to follow preventive dentistry principles to ensure the best oral health maintenance for their patients, as prevention is a key component of public health initiatives. This study aims to assess the knowledge and attitudes toward preventive dental care among Iraqi dentists and senior dental students.

Methodology: A cross-sectional study, conducted from June to August 2023, involved 876 Iraqi dentists and 587 senior dental students in Baghdad, utilizing a physically paper-based self-administered questionnaire. Participants' level of knowledge was assessed based on a 5-point Likert scale to 12 statements on preventive dental care. Their attitudes were inquired by selecting one option from a 7-point Likert scale on four pairs of bipolar adjectives describing preventive dentistry. Higher scores were considered more accurate knowledge and more positive attitudes. Statistical evaluation included the Chi-square test and regression analysis.

Results: In total, data from 841 dentists and 567 students were analyzed (response rate: 96%). Around 90% of dentists (N=745) and students (N=502) acknowledged the impact of sugar, sealant, and water fluoridation on dental caries, but about 80% of dentists (N=662) and students (N=446) undervalued the role of fluoridated toothpaste. Women (OR=1.4, 95% CI: 1.02-2.1) and dentists, within one year of their graduation (OR=1.9, 95% CI: 1.1-3.5), exhibited higher levels of knowledge. More favorable attitudes towards preventive dentistry were associated with a higher level of knowledge among dentists (OR=1.6, 95% CI: 1.2-2.3). Regarding dental students, those from private dental schools showed higher scores of knowledge than their counterparts from public schools (OR=2.1, 95% CI: 1.3-3.4). The majority of participants held the belief that preventive dentistry is beneficial for the community but just about 60% of dentists (N=477) and students (N=300) exhibited a positive attitude toward the economic advantages of preventive dentistry for dentists, as well as the ease of engaging in preventive dental practices.

Conclusions: The dentists and dental students in this study demonstrated satisfactory knowledge and a favorable attitude toward most aspects of preventive dentistry. However, deficiencies were noted in certain areas, such as the application of topical fluoride for preventing dental caries, as well as a substantial proportion of individuals who lacked a positive perspective on the economic benefits of preventive dentistry. Hence, there is a clear need for educational interventions during their undergraduate training and postgraduate continuing education to enhance their knowledge levels and cultivate a more positive attitude towards preventive dentistry.

## Introduction

Dental caries remains a prevalent public health issue worldwide, contributing to a considerable burden on individuals and healthcare systems [1]. Dental caries frequently lead to pain, discomfort, and the risk of tooth loss, with notable social and economic consequences that affect individuals' quality of life [2]. Studies consistently associate tooth loss with reduced health-related quality of life, affecting functions like eating, speaking, and socializing [3], leading to lower levels of satisfaction with oral health and overall well-being. Based on a recent report from the World Health Organization (WHO), oral diseases impact nearly 3.5 billion individuals globally, which includes approximately two billion people with tooth decay in their permanent teeth and more than half a billion children with caries in their primary teeth, the majority of them from middle-income nations [4]. These statistics underscore the pressing need to shift the focus of dental services toward prevention, aligning with the recommendations of the WHO [5]. This paradigm shift requires primarily knowledgeable dental care providers with positive attitudes towards the prevention of oral diseases who believe in preventive dentistry as a proactive approach and adopt it in their practice to tackle oral health challenges.

Acquiring knowledge and developing a mindset are essential for the delivery of optimal dental services [6]. Profound knowledge enables dental care providers to comprehend preventive measures and to instruct patients on better practice of oral hygiene. Moreover, a favorable attitude toward preventive dentistry encourages professionals to prioritize prevention and to embrace strategies for promoting oral health. Research has demonstrated that a high level of knowledge and a positive attitude toward preventive dentistry are correlated with enhanced proficiency in delivering preventive care among dentists [7] and dental students [8].

Dental care providers are responsible for acquiring and continuously improving their professional knowledge and attitude. Assessing their knowledge and attitude towards preventive dental care is crucial as it can directly impact public health outcomes by promoting early intervention and reducing the burden of dental diseases [9]. Studies suggest varying levels of knowledge and attitudes regarding preventive dental care among dental students and dentists. For example, in two studies in Indonesia [10] and Nepal [11], dental students exhibited low levels of knowledge in certain aspects of preventive dentistry, whereas dental students in Saudi Arabia [12], Yemen [13], and Iran [14] demonstrated high knowledge and attitude in this regard. Variation also exists in dentists' knowledge and attitudes. For instance, participants from different studies from the United Kingdom [15], India [16], and Iraq [17] displayed limited knowledge of specific preventive dental domains, whereas their counterparts from Saudi Arabia [18] and India [19] showed adequate knowledge and a positive attitude.

In Iraq, with a dentist/population ratio of 2.6/10,000 and a great desire for an expanding supply of dentists [20], the prevalence of dental caries is relatively high since at least one out of three individuals experience untreated dental caries [21]. While certain investigations propose a distinct correlation between the dental workforce and the dental health status within a community, conflicting findings are put forth by other studies, resulting in a contentious debate within the discipline [22]. Nevertheless, as an ethical and professional responsibility, dentists are expected to play their role in the prevention of oral diseases both for their individual patients and as advocates for health promotion in society [23]. Despite great emphasis on educating dentists who are equipped with preventive and community orientation [24], the level of adherence to this recommendation is unclear [25].

Recent studies showed that the majority of Iraqi dentists follow traditional invasive, non-conservative approaches during their practices, which was attributed to their undergraduate education [26,27]. Another study also demonstrated insufficient knowledge concerning the role of fluoridated toothpaste and the utilization of dental explorers in diagnosing dental caries and a lack of enthusiasm for preventive dentistry [28]. The primary objective of this study was, therefore, to assess the attributes of knowledge and attitudes among Iraqi dentists and senior dental students regarding preventive care.

## Materials and methods

Subjects and sampling issues

This human observational study conforms to the STROBE Guidelines [29]. Subjects for the present cross-sectional study were 3,160 Iraqi dentists working in public health facilities in Alkarkh Baghdad health directorate, and 2,442 senior dental students attending state and private dental schools in Baghdad from June to August 2023. The sample size was determined using the following formula [30]: n = nօ/[1 + (nօ -1/N)]: in which “n” is the required sample size, nօ = Z2 (P) (1-P) / d2; “Z” the level of confidence = 1.96, “N” is the size of population from which the sample is recruited (provided by the committee of deans of colleges of dentistry in Iraq and Baghdad health directorate, “P” is estimated proportion of the population that presents the characteristic (here the proportion of dentists with high knowledge scores according to a previous study [31] = 0.59, and “d” is maximum allowed error = 0.059 (0.1 multiplied by P).

According to the Iraqi law for apprenticeship of medical and health professionals [32], after graduation, each dentist must work as a “trainee” at specialized public dental health centers under the supervision of specialist dentists. This stage is followed by two years of “apprenticeship” in which the dentists work at the public primary health centers in suburban and rural areas. After these two years, the dentist fulfills the requirements of medical apprenticeship and becomes a practitioner who is eligible to move to work in urban public healthcare centers as a general dental practitioner (GDP). To include all types of dentists working in public health services, based on the formula mentioned above, 201 trainees, 217 apprentices, and 223 GDPs were considered a sample size for this study. 

In Baghdad, there are two health directorates with relatively similar conditions in terms of the number and distribution of health centers. For this study, the Alkarkh health directorate was randomly selected (lottery method). The inclusion of health centers in the study was determined using a simple random sampling (lottery method). Five out of eight specialized public dental centers (for trainees), 22 out of 73 suburban centers (for apprentices), and 40 out of 60 public primary health centers (for GDPs) within the Alkarkh health directorate were randomly chosen. A convenient sample of dentists was then selected from these chosen centers for participation in the study.

Regarding dental students, three out of four state dental schools and five out of 13 private dental schools in Baghdad were selected by simple random selection (lottery method). The selected schools were visited one by one in order to reach the estimated sample size of 190 dental students from the state and 236 dental students from private dental schools. A convenient sample of students was then recruited from the selected schools for inclusion in the study. The data collection continued to reach the predetermined number of participants in the sample size calculation.

Participant recruitment

The participants were met in their working settings and classrooms, where they were invited to (physically) participate voluntarily in filling out the paper-based questionnaire, which typically required approximately 20 minutes to complete. The completed questionnaires were collected immediately from students and within two days from the dentists. A lottery for 10 high-speed handpieces was offered as an incentive to encourage participation.

Research instrument

This study used an updated version of a self-administered paper-based questionnaire adapted from similar studies [31,33]. The participants answered an English language questionnaire as it is the formal language in the higher education system in Iraq (Appendix 1). To assess the validity of the questionnaire, a group of eight professors from various departments of the School of Dentistry, Shahid Beheshti University of Medical Sciences, and Mustansiriyah University were consulted to provide their expert opinions. The professors evaluated the questionnaire's statements in terms of writing style, clarity, and fluency to determine its face validity. Regarding content validity, the content validity ratio (CVR) and content validity index (CVI) were calculated. The professors were asked to choose one option from the scale of “necessary,” “useful but unnecessary,” and “unnecessary” to establish the CVR using the formula: CVR = [(Ne - (N/2))/(N/2)], that took into account the number of evaluating experts (i.e., eight professors denoted by “N”) and the number of experts who chose the “necessary” option for each question (denoted by “Ne”). Furthermore, items with CVRs below 0.75 were eliminated based on the Lawshe table [34]. To calculate CVI, the professors assessed the relevance, clarity, and simplicity of each statement in the questionnaire using a 4-point Likert scale. The relevance was rated as “quite relevant,” “relevant,” “somewhat relevant,” and “irrelevant,” while clarity and simplicity were rated as “very good,” “good,” “weak,” and “very weak.” The CVI was then calculated as the ratio of professors who selected the options “quite relevant” and “relevant” for relevance and “very good” and “good” for clarity and simplicity to the total number of professors who provided feedback on that particular item. Items with CVI values below 0.8 were subsequently removed from the analysis [34-36]. The expert's ratings led to the omission of three out of 15 knowledge items and one out of five attitude items.

The questionnaire reliability was assessed using (test-retest) method [37]. Twenty students and 20 dentists completed the questionnaire twice at a two-week interval [38]. Based on their responses, the weighted kappa was computed for each item of the questionnaire; the results were within the acceptable range (0.4-0.9) [39].

The final questionnaire included several sections, capturing participants' background information such as age and gender. For dentists, additional information included their classification as trainee, apprentice, or GDP, the number of years of experience in dentistry, working sector, and their engagement in continuing education (CE) courses related to caries prevention, along with their interest in participating in future courses. For dental students, the questionnaire gathered information about whether one of their parents is a dentist, their desire to join CE courses in caries prevention, and whether they were studying in a state or private dental school.

Assessment of participants' knowledge of preventive dental care

Twelve statements covering different aspects of caries prevention were used to assess respondents' knowledge of preventive dental care. They answered anonymously on a 5-point Likert scale ranging from fully agree (score 5) to fully disagree (score 1). The scores were then summed up to determine each participant's knowledge score (theoretically ranging from 12 to 60). Participants' level of knowledge was categorized into high (≥48; if options “fully agree” or “agree” were selected for all 12 knowledge statements) and low (<48; in case options “don’t know,” “disagree,” or “fully disagree” were selected for all 12 knowledge statements) categories.

Assessment of participants' attitude towards preventive dental care

In order to assess the respondents' attitudes toward preventive dentistry, a 7-point semantic differential scale of four qualities and their opposites was used. Answering options for each of the paired qualities were given scores from one to seven, with the higher scores for the more favorable attitudes resulting in the possible attitude scores ranging from a minimum of 4 to a maximum of 28. For further analysis, attitude scores were dichotomized into high positive (≥24; if scores 6-7 were selected for all bipolar qualities) and low (<24, if scores 1-5 were selected for all bipolar qualities).

Ethical considerations

The research has received approval from the Ethics Committee of Shahid Beheshti School of Dentistry (ethics code: IR.SBMU.DRC.1402.007). The questionnaire was completed anonymously, and all respondents were assured of the confidentiality of their responses. Their agreement to complete the questionnaire was considered as an expression of informed consent to participate in the study.

Statistical evaluation

The statistical evaluation included the Chi-square test to assess differences in frequencies. Following the bivariate analysis, multicollinearity was examined among all independent variables. Logistic regression models, using the enter method, were applied to the data to assess the factors associated with knowledge and attitude. The corresponding odds ratio (OR) and 95% confidence intervals (95% CI) were defined for the likelihood of having high knowledge and positive attitude. The p-value was set at < 0.05. The Statistical Package for the Social Science (Version 27.0; IBM Corp., Armonk, NY, 2020) was used to perform statistical tests. For further analysis, cases with more than 20% missing values were excluded (six dentists for the knowledge section) and (38 dentists and 58 students for the attitude section) [40]; otherwise, they were substituted by the item mean value.

## Results

**Figure 1:**
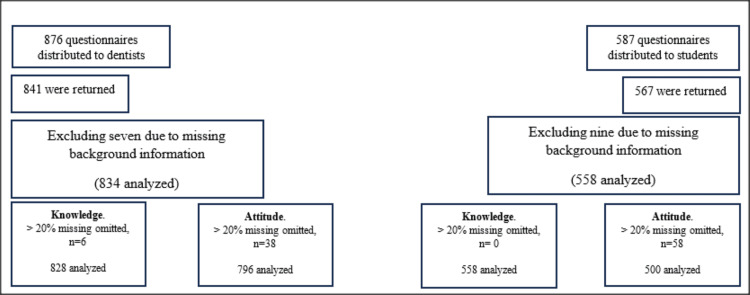
Flowchart indicating the number of participants included in the final analysis Data are represented as numbers (n).

Of 876 questionnaires distributed to dentists, 841 were returned (96% response rate). After excluding seven due to missing background information, 834 were analyzed. Among dental students, 567 out of 587 distributed questionnaires were returned (96% response rate), with nine exclusions due to missing background information, resulting in 558 cases for final analysis (Figure 1).

In this study, the participants' mean age was 29 ± 6.2 years for dentists and 23 ± 1.3 years for students. The dentists had varying levels of experience, ranging from 1 to 40 years. The participants' background information is presented in Table 1. No statistically significant differences in gender and eagerness to attend continuous education courses were observed between dentists and dental students. Around one out of five of the participants (165 dentists and 111 students) were categorized as having a high level of knowledge regarding the prevention of dental caries, and more than 40% of them (318 dentists and 200 students) showed a highly positive attitude towards preventive dentistry. No statistically significant differences in knowledge and attitude scores were observed between dentists and dental students (Table 2).

**Table 1:** Distribution of the Iraqi dentists (n=834) and senior dental students (n=558) according to their background information. Data are represented as numbers (percentage) n (%).

Dentists	n (%)
Gender	
Men	283 (34)
Women	551 (66)
Dentists' category	
Trainee	307 (37)
Apprentice	263 (32)
General dental practitioner	264 (32)
Working sector	
Public	499 (60)
Public and private	335 (40)
Attended continuing education courses	
Yes	111 (13)
No	723 (87)
Like to attend continuing education courses	
Yes	552 (66)
No	282 (34)
Students	n (%)
Gender	
Men	193 (35)
Women	365 (65)
Dental School	
State	227 (41)
Private	331 (59)
One or both parents' dentists	
Yes	27 (5)
No	531 (95)
Like to attend continuing education courses	
Yes	388 (70)
No	165 (30)

**Table 2:** Percentages of Iraqi dentists (n= 843) and senior dental students (n=558) belonging to the category of high levels of knowledge of and attitude towards preventive dentistry. ^1^By Chi-square test. Data are represented as percentages (%). Statistical significance was defined as p<0.05.

	Knowledge		P-value^1^	Attitude		P-value^1^
	High (%)	Low (%)		High (%)	Low (%)	
Dentists	21	79	0.3	42	58	0.8
Students	23	77		42	58	

**Figure 2:**
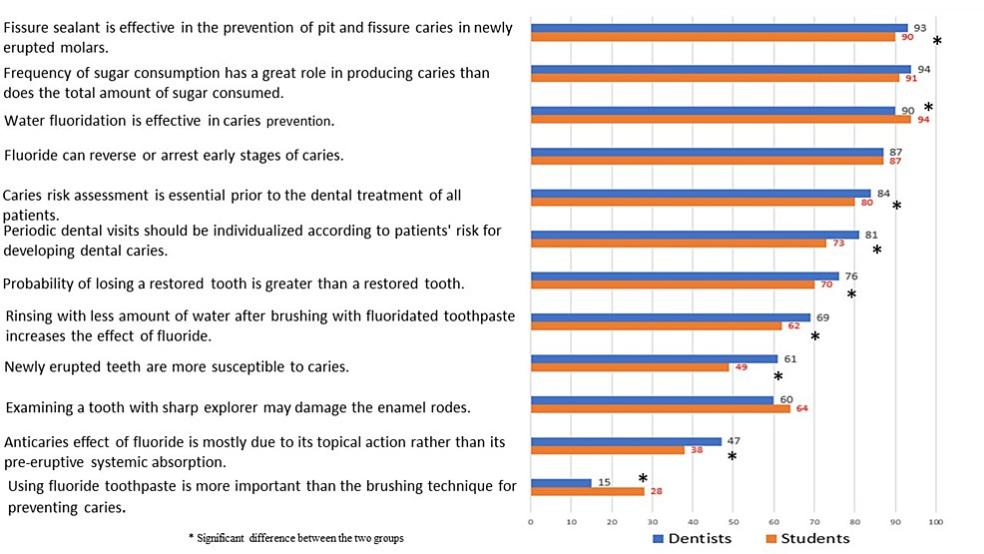
The percentage of Iraqi participants agreed (fully agree and agree) with statements on the prevention of dental caries. Data are represented as percentage (%). Statistical significance was defined as p<0.005.

**Figure 3:**
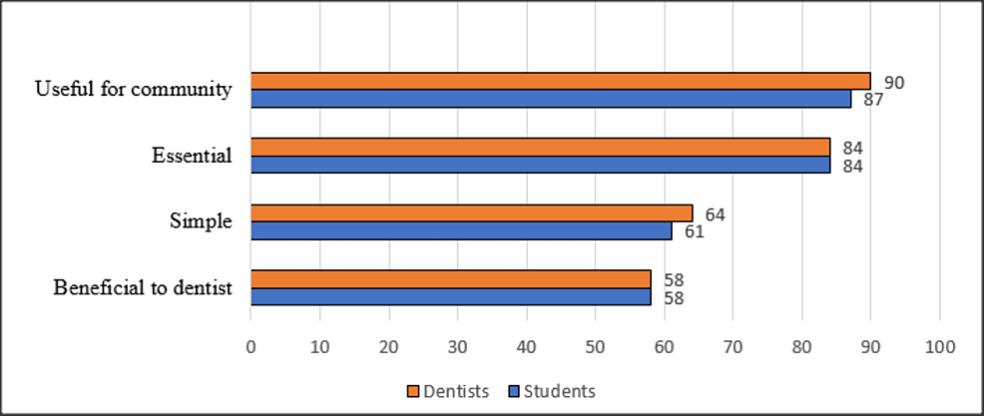
The percentage of Iraqi participants who characterized preventive dentistry by selecting scores 5, 6, or 7 from a 7-point Likert scale. Data are represented as percentage (%). Statistical significance was defined as p<0.005.

As it appears in Figure 2, over 90% of participants (745 dentists and 502 students) believed in the caries-preventive efficacy of fluoridated drinking water, limiting the frequency of using sugary food or drinks, and fissure sealant application. Fewer than 20% of dentists (N=165) and 30% of students (N=167) attributed higher value to fluoridated toothpaste than brushing techniques for caries prevention. The majority of the respondents characterized preventive dentistry as “useful for the community” and an “essential” subject (Figure 3). 

Determinants of the dentists' and students' knowledge and attitude regarding preventive dentistry as assessed by four separate binary logistic regression models are presented in Tables 3, 4. When controlling for other factors, women (OR=1.4, 95% CI: 1.02-2.1) and a trainee dentist (OR=1.9, 95% CI: 1.1-3.5) were significantly associated with a higher level of knowledge. Moreover, dentists with higher levels of knowledge were more likely to have a high positive attitude toward preventive dentistry (OR=1.6, 95% CI: 1.2-2.3). For students, studying in a private rather than a state dental school was significantly associated with a higher level of knowledge (OR=2.1, 95% CI: 1.3-3.4).

**Table 3:** Determinants of knowledge of and attitude towards preventive dentistry among Iraqi dentists using regression analysis. *General Dental Practitioner, § Continuous education (CE), # Crude odds ratio, †The p-value associated with the crude odds ratio, $Adjusted odds ratio, ‡The p-value associated with the adjusted odds ratio, *95% Confidence Intervals, ¶Reference group. Data are represented as Odds Ratios (OR) with 95% Confidence Intervals (95% CI). Statistical significance was defined as p<0.05. Adjusted odds ratios (OR$) account for potential confounders.

		OR^#^	95% CI^*^	P-value^†^	OR^$^	95% CI^*^	P-value^‡^
High knowledge							
Age (years)		0.9	0.9-1.0	0.07	0.9	0.9-1.0	0.9
Gender	Men	Ref.^¶^					
	Women	1.2	0.8-1.7	0.20	1.4	1.02-2.1	0.003
Dentists' category	GDP^*^	Ref.^¶^					
	Trainee	1.6	1.1-2.5	0.01	1.9	1.1-3.5	0.01
	Apprentice	1.3	0.9-2.1	0.14	1.3	0.8-2.3	0.2
Working setting	Public	Ref.^¶^					
	Public and Private	0.9	0.6-1.2	0.6	1.3	0.9-1.8	0.1
Attended CE^§ ^courses	No	Ref.^¶^					
	Yes	0.9	0.5-1.4	0.6	0.8	0.5-1.4	0.5
Like to attend CE courses	No	Ref.^¶^					
	Yes	1.1	0.8-1.6	0.4	1.1	0.8-1.6	0.3
High positive attitude							
Age (years)		0.99	0.9-1.0	0.7	0.9	0.9-1.0	0.4
Gender	Men	Ref.^¶^					
	Women	1.10	0.8-1.4	0.5	1.07	0.7-1.4	0.6
Dentists' category	GDP^*^	Ref.^¶^					
	Trainee	0.9	0.6-1.2	0.5	0.8	0.5-1.2	0.3
	Apprentice	0.9	0.6-1.3	0.8	0.8	0.5-1.3	0.4
Working setting	Public	Ref.^¶^					
	Public and Private	0.9	0.6-1.2	0.5	1.1	0.8-1.5	0.5
Attended CE^§^ courses	No	Ref.^¶^					
	Yes	0.9	0.6-1.5	0.9	0.9	0.6-1.4	0.8
Like to attend CE courses	No	Ref.^¶^					
	Yes	1.3	0.9-1.8	0.06	1.3	0.9-1.8	0.07
Knowledge	Low	Ref.^¶^					
	High	1.6	1.2-2.3	0.002	1.6	1.2-2.3	0.002

**Table 4:** Determinants of knowledge of and attitude towards preventive dentistry among Iraqi senior dental students using regression analysis. *General Dental Practitioner, § Continuous education (CE), # Crude odds ratio, †The p-value associated with the crude odds ratio, $Adjusted odds ratio, ‡The p-value associated with the adjusted odds ratio, *95% Confidence Intervals, ¶Reference group. Data are represented as Odds Ratios (OR) with 95% Confidence Intervals (95% CI). Statistical significance was defined as p<0.05. Adjusted odds ratios (OR$) account for potential confounders.

		OR^#^	95% CI^*^	P-value^†^	OR^$^	95% CI^*^	P-value^‡^
High knowledge							
Gender	Men	Ref.^¶^					
	Women	1.2	0.7-1.8	0.3	1.1	0.7-1.7	0.6
Dental school	State	Ref.^¶^					
	Private	2.1	1.3-3.3	< 0.001	2.1	1.3-3.3	0.001
A parent is a dentist	No	Ref.^¶^					
	Yes	0.2	0.06-1.2	0.09	0.2	0.06-1.1	0.07
Like to attend CE^§^ courses	No	Ref.^¶^					
	Yes	1.3	0.8-2.1	0.2	1.1	0.6-1.8	0.6
High positive attitude							
Gender	Men	Ref.^¶^					
	Women	1.4	0.9-2.1	0.05	1.4	0.9-2.1	0.05
Dental school	State	Ref.^¶^					
	Private	1.09	0.7-1.5	0.6	1.1	0.7-1.6	0.5
A parent is a dentist	No	Ref.^¶^					
	Yes	0.7	0.3-1.9	0.5	0.8	0.3-2.1	0.7
Like to attend CE^§^ courses	No	Ref.^¶^					
	Yes	0.9	0.6-1.3	0.7	0.8	0.5-1.2	0.4
Knowledge	Low	Ref.^¶^					
	High	1.4	0.9-2.1	0.1	1.4	0.9-2.1	0.1

## Discussion

The present findings indicated that a significant proportion of Iraqi dentists and dental students possess a general understanding of the impact of sugar, sealants, and water fluoridation on dental caries; however, they undervalue the role of topical fluoride. Their inclination toward preventive dental care is predominantly expressed through acknowledging its usefulness for the community and recognizing it as an indispensable discipline.

Preventive dentistry is usually an essential subject in most of the undergraduate dental curriculum [41]. The competency received by dental students out of these curricula could vary [42-44]. For Iraqi senior dental students, the results suggest that the Iraqi curriculum effectively highlights the primary means of preventing caries [45,46]. However, despite the inclusion of preventive dentistry as a dedicated topic during the final year of the student's curriculum and the integration of various aspects of preventive measures throughout different subjects such as community dentistry, operative dentistry, pedodontics, oral pathology, and oral medicine, the percentage of students demonstrating a high level of knowledge in preventive dentistry remains low (21%, N=117). This deficiency in knowledge, particularly concerning the significance of topical fluoride, is consistent with the findings of similar questionnaire-based studies conducted on dental students in Iran [47], Indonesia [10], Yemen [13], and Nigeria [48] in which 15%-35% of students reported optimal level of knowledge. Given that dentists and dental students demonstrate a higher enthusiasm towards training focused on clinical procedures like aesthetic restorations, implants, and root canal treatments [49,50], there is a need to underscore fundamental subjects such as prevention and employ innovative educational approaches to convey pertinent concepts. Educational interventions have proven to be successful in enhancing the knowledge and improving the attitude of dentists and dental students about topics such as ergonomics [51] and the prevention of papillomavirus infection [52].

The visual-tactile method, a common technique in clinical dentistry, involves using a sharp probe for detecting dental caries [53]. However, the improper application of this method, which entails correlating the presence of caries lesions with the resistance to withdrawal of a sharp probe when forcefully inserted into suspected pits or fissures, has raised concerns [54]. This misguided practice not only increases the risk of enamel damage but also exacerbates the issue of overtreatment [55]. Despite the inclusion of information about these risks in the dental curriculum, there appears to be a limited understanding among students. This lack of comprehension may be influenced by the practices of clinical instructors. To address this issue, it is crucial to bridge the gap between theoretical knowledge and clinical practice in the training of students [56].

The student's attitude toward prevention plays a vital role in shaping their future profession and influencing their decision to implement preventive dental care [57]. The majority of the studied students conveyed a positive attitude toward the societal advantages of preventive dentistry. Nevertheless, a significant number of students shared the belief that prevention does not result in financial gains, in line with the viewpoints of Turkish [8] and Iranian students [58] in questionnaire-based studies, which indicates that many students are concerned about the economic aspects of practicing preventive approaches. Consequently, this skepticism may contribute to a potential disregard for offering preventive treatments.

Private dental school students exhibited significantly higher odds of falling into the high knowledge category than their state dental school counterparts. Notably, despite the uniformity of the dental curriculum, students in private dental schools might be influenced by their experienced professors, many of whom are retired dental professionals with extensive expertise.

In order to ensure that clinical practice remains up-to-date with the advancements in dental science, practitioners must enhance their understanding and attitudes towards different care options. By possessing accurate knowledge, dentists will be empowered to make informed and suitable decisions about the health of their patients. In this study, aligning with findings from questionnaire-based studies in Iraq [17], Iran [31,33], and India [19], the majority of dentists showed adequate knowledge regarding the influence of sugar, sealants, and water fluoridation on dental caries, with noticeable gaps identified in their comprehension of the preventive role of topical fluoride, as evidenced by their undervaluation of its significance. These observations contradict the belief held by a panel of experts that fluoride indeed plays a crucial role in the prevention of dental caries [59] and misalign with dentists from Finland [60] and Scandinavian countries [61], who recognized different forms of fluoride as essential measures for preventing tooth decay, which could potentially be a contributing factor to the observed reduction in dental caries rates in these regions [62].

The reduction of post-brushing water rinsing could maximize the potential benefits of fluoride present in toothpaste [63]. The results reflected a lack of awareness among dentists in the present study regarding the significance of this oral health message. Likewise, similar inadequacies have been reported from dentists in South Africa [64] and Iran [14,31,33], which may be attributed to the absence of this information in the dental curriculum and the influence of prevalent social norms, as evidenced by the common cultural practice of thorough rinsing after brushing [65].

The study's findings showed that dentists generally recognize the benefits of preventive dental care for the community, suggesting their willingness to engage actively in preventive measures. However, the dentists' responses also indicate a lack of economic viability associated with preventive dental care. This aligns with similar findings from studies conducted among Libyan [66] and Australian [67] dentists. This highlights the need for policies that promote preventive interventions in the dental healthcare delivery system. One strategy might be to encourage dentists to provide these services by adjusting pricing for preventive dental care [68] or changing how they are reimbursed for these treatments [69].

The response patterns to various knowledge statements exhibited remarkable similarities between dentists and students. Furthermore, the disparity in their total knowledge scores did not demonstrate statistical significance compared to that of students. This may suggest that dentist's knowledge has not improved or evolved after graduation. This observation may reflect a limited inclination among dentists toward preventive dentistry that may stem from perceived barriers in their practice, such as inadequate reimbursement, time constraints due to high demand for curative care, and patients' reluctance to invest in preventive measures [70,71]. Additionally, it could be attributed to the absence of a professional obligation to participate in CE programs as part of the license renewal for Iraqi dentists [72]. Within the same context, the data revealed that only one out of ten dentists participated in CE courses about caries prevention, with little impact on their knowledge or attitude. On the other hand, more than half of the respondents expressed eagerness to attend such courses in the future. This could suggest their enthusiasm for the field of preventive dentistry and their willingness to engage in associated scientific meetings, should they occur.

It is worth noting that women in this study showed higher knowledge scores than men. Moreover, the higher odds of newly graduated dentists being in the high knowledge category may specifically be attributed to the recency of their graduation, aligning with the findings of British [15] and Korean [73] dentists. Dentists with higher knowledge scores presented highly positive attitudes, highlighting the pivotal role of knowledge in shaping dentists' perspectives and influencing their professional conduct.

The study benefits from a random selection of the subjects' settings, a high response rate, and the use of a validated questionnaire. These factors enhance the generalizability and reliability of the collected data. The study, however, recognizes inherent limitations in questionnaire surveys, noting that self-reported data on participants' knowledge and attitudes towards preventive dentistry may introduce response bias due to social desirability or inaccurately recalled information. Furthermore, while logistic regression models were utilized to analyze factors related to knowledge and attitude, the study's observational design restricts causal inference, potentially influenced by unmeasured confounding variables.

## Conclusions

The dentists and dental students in this research exhibited satisfactory levels of knowledge and displayed a favorable attitude toward most aspects of preventive dentistry. This finding holds promise as it suggests the potential for them to implement preventive dentistry principles in their practice. Nevertheless, the inadequate knowledge in certain areas, notably regarding the application of topical fluoride for dental caries prevention, as well as a significant percentage of dentists and students who expressed concerns about the viability of preventive dentistry, underscores the necessity for a more robust focus on this subject within dental education. Consequently, enhancing both knowledge acquisition and attitude adjustment among current and prospective dental professionals with regard to preventive dentistry could be achieved through targeted educational initiatives within the undergraduate dental curriculum and postgraduate CE.

## Appendices

Appendix 1

**Table 5:** Questionnaire

	The Questionnaire			
1. Background information				
1.1. Gender:	1. Male	2. Female		
1.2. Year of birth…….				
1.3. Do you study in (1. State or 2. Private) dental school?				
1.4. Dental school name ………..				
1.5. Is one of your parents a dentist?		1. Yes	2. No	(for students)
1.6. You are a -------------				
1. Trainee dentist.				
2. Apprentice dentist				
3. General dental practitioner (GDP)				
1.7. Time passed since your graduation …… years				
1.8. Do you work in				
a. public sector only.				
b. public and private sectors.				
1.9. Have you ever attended a continuous education course on dental caries prevention?				
	1. No	2. Yes.		
1.10. Do you like to attend a continuous education course on dental caries prevention?				
	1. No	2. Yes.		
2. Knowledge of caries prevention				
2.1. Fluoridation of drinking water is an effective way to prevent dental caries.				
1. Strongly agree				
2. Agree				
3. Disagree				
4. Strongly disagree				
5. Don't know				
2.2. The frequency of sugar consumption has a greater role in producing caries in comparison with the total amount of sugar consumed at once.				
1. Strongly agree				
2. Agree				
3. Disagree				
4. Strongly disagree				
5. Don't know				
2.3. Fissure sealant is effective in the prevention of pit and fissure caries in newly erupted molars.				
1. Strongly agree				
2. Agree				
3. Disagree				
4. Strongly disagree				
5. Don't know				
2.4. Compared to a sound tooth, the probability of losing a restored tooth is greater.				
1. Strongly agree				
2. Agree				
3. Disagree				
4. Strongly disagree				
5. Don't know				
2.5. Rinsing teeth with a less amount of water after tooth-brushing with fluoridated toothpaste increases the effect of fluoride.				
1. Strongly agree				
2. Agree				
3. Disagree				
4. Strongly disagree				
5. Don't know				
2.6. Examining a newly erupted tooth with a sharp explorer may damages the enamel rods and makes the tooth vulnerable to caries.				
1. Strongly agree				
2. Agree				
3. Disagree				
4. Strongly disagree				
5. Don't know				
2.7. Using fluoride toothpaste is more important than the brushing technique for preventing caries.				
1. Strongly agree				
2. Agree				
3. Disagree				
4. Strongly disagree				
5. Don't know				
2.8. Caries risk assessment is essential prior to the dental treatment of all patients.				
1. Strongly agree				
2. Agree				
3. Disagree				
4. Strongly disagree				
5. Don't know				
2.9. Periodic dental visits should be individualized according to patients’ risk for developing dental caries				
1. Strongly agree				
2. Agree				
3. Disagree				
4. Strongly disagree				
5. Don't know				
2.10. Fluoride can reverse or arrest early stages of caries.				
1. Strongly agree				
2. Agree				
3. Disagree				
4. Strongly disagree				
5. Don't know				
1. Strongly agree				
2. Agree				
3. Disagree				
4. Strongly disagree				
5. Don't know				
2.11. Newly erupted teeth are more susceptible to caries.				
1. Strongly agree				
2. Agree				
3. Disagree				
4. Strongly disagree				
5. Don't know				
2.12. Anticaries effect of fluoride is mostly due to its topical action rather than its pre-eruptive systemic absorption.				
1. Strongly agree				
2. Agree				
3. Disagree				
4. Strongly disagree				
5. Don't know				
3. Attitudes towards preventive dentistry				
Please describe your opinion about preventive dentistry (in general) on the following scale of four qualities and their opposites. For example, if you think preventive dentistry is completely beneficial, circle number 7 and if you think it is completely costly, circle number 1. Otherwise, circle an appropriate medial number.				
Costly for dentist	1…2...3…4…5…6…7		Beneficial to dentist	
Useless for community	1…2...3…4…5…6…7		Useful for community	
Non-essential	1…2...3…4…5…6…7		Essential	
Difficult	1…2...3…4…5…6…7		Simple	
